# Multiple sclerosis patients’ response to COVID-19 pandemic and vaccination in Egypt

**DOI:** 10.1186/s41983-022-00573-8

**Published:** 2022-11-17

**Authors:** Adel Hassanein Elsayed Gad, Sandra Mohamed Ahmed, Mostafa Yahya Abdelmohsen Garadah, Ahmed Dahshan

**Affiliations:** Cairo University, Cairo, Egypt

## Abstract

**Background:**

On 11 March 2020, WHO declared COVID-19 has become a pandemic. This had an impact on everyday activity for every person. For special groups such as multiple sclerosis patients, the situation is a little bit confusing. In this study, COVID-19 infection impact on MS patients, willingness for vaccination, percentage of vaccinated patients and adverse effects of different vaccines were investigated. This cross-sectional descriptive study included 160 Egyptian MS patients. Demographic and clinical characteristics of all patients were extracted from their files MS unit archives. All these patients were contacted either by telephone and an oral informed consent was taken or in-person on their scheduled follow-up and informed written consent was taken to join this study. Patients were asked about: COVID-19 infection, severity of infection, and vaccination using a special questionnaire developed by the authors.

**Results:**

Only 39 (24.3%) patients have had COVID-19 infection with confirmed diagnosis. Most of infected patients (84.6%) were treated at home with no need for hospital admission. Five patients (12.8%) reported symptom suggestive of relapses after COVID-19 infection. Sixty-five patients (40.6%) were vaccinated against COVID-19. Out of these vaccinated patients, 22 patients (33%) developed adverse events from vaccine. These adverse events were self-limiting and related to local injection site and general manifestations. MS relapse after vaccination was reported in 7.7% of the vaccinated group.

**Conclusion:**

Prevalence of COVID-19 infection and severity of infection were equal to general population. Risk of relapse is low either with infection or vaccination. No severe adverse events were reported after vaccination.

## Background

From the first cases reported in China in December 2019, a form of severe acute respiratory distress syndrome due to SARS-CoV-2, a new human-infecting beta-coronavirus, became a global health threat. Notably, the clinical spectrum of COVID-19 is broad, COVID-19 patients presented with a varied range of symptoms from asymptomatic to severe respiratory failure [1]. Patients with MS (PwMS) are special group who has a chronic condition and receive treatments that affect the immunity and makes them more prone to infection. There is still concern among neurologists regarding their morbidity and mortality with COVID-19 [2]. Also, there is a lot of insecurity about the safety and efficacy of SARS-CoV-2 vaccination among MS patients [3]. In this study, we tried to fill gaps regarding characteristics of COVID-19 infection in PwMS and patients’ response to vaccination.

## Methods

This was a cross-sectional descriptive study that included 160 Egyptian MS patients in 2021 during the peak of COVID-19 pandemic. We included all MS patients who were diagnosed according to revised McDonald criteria 2017 [4]**.** We excluded patients above 65 years, and patients with comorbid diseases as diabetes, hypertension, bronchial asthma or any other chronic illness which could affect immune system and increase the risk of COVID-19 infection. The study was approved by the ethical committee. Patients provided informed written or oral consents for participation. Regarding patients less than 18 years, their legal guardians gave the consent.

A special questionnaire was designed by the authors to collect data. This questionnaire included the following information: name, age, gender, telephone number, any other comorbidities other than MS, smoking, type of MS (RRMS, SPMS, PPMS), type of current disease modifying drug (DMD), Compliance on DMD, If DMD is stopped and why, history of COVID-19 infection (when, symptoms, severity, diagnosis, plan of management, post-infection symptoms, and if any relapses were associated with the infection), before vaccination (willingness to receive vaccination, source of information, your doctor’s consultation), COVID-19 vaccination history (which type, any adverse events, for how long lasted the adverse events, any associated relapses). Patients were interviewed either in-person during regular clinic visit or by telephone (50 patients in-person and 110 patients by telephone). Patients were asked to submit all documents related to the infection, including laboratory tests—CBC, CRP, D-dimer, ferritin, LDH, RT-PCR swab results for SARS COV-2, CT scans of the lungs, and MRI brain. These documents were reviewed to be sure about diagnosis of COVID-19. The positive PCR result or compatible lung CT scan was acceptable for COVID-19 diagnosis. Demographic and clinical characteristics of all patients were extracted from their files MS unit archives. Data were expressed as mean ± SD for quantitative variables and counts (%) for categorical variables. We used SPSS 21 (SPSS, Chicago, IL, USA) for the statistical analysis of data. Between-group differences were analyzed by Student's t-test for quantitative variables. The association of categorical variables were tested by Chi-square or Fisher's exact tests, where indicated using the statistical package for social science (SPSS) version 22 (SPSS, Armonk, New York: International Business Machines Corporation).

## Results

**Fig. 1:**
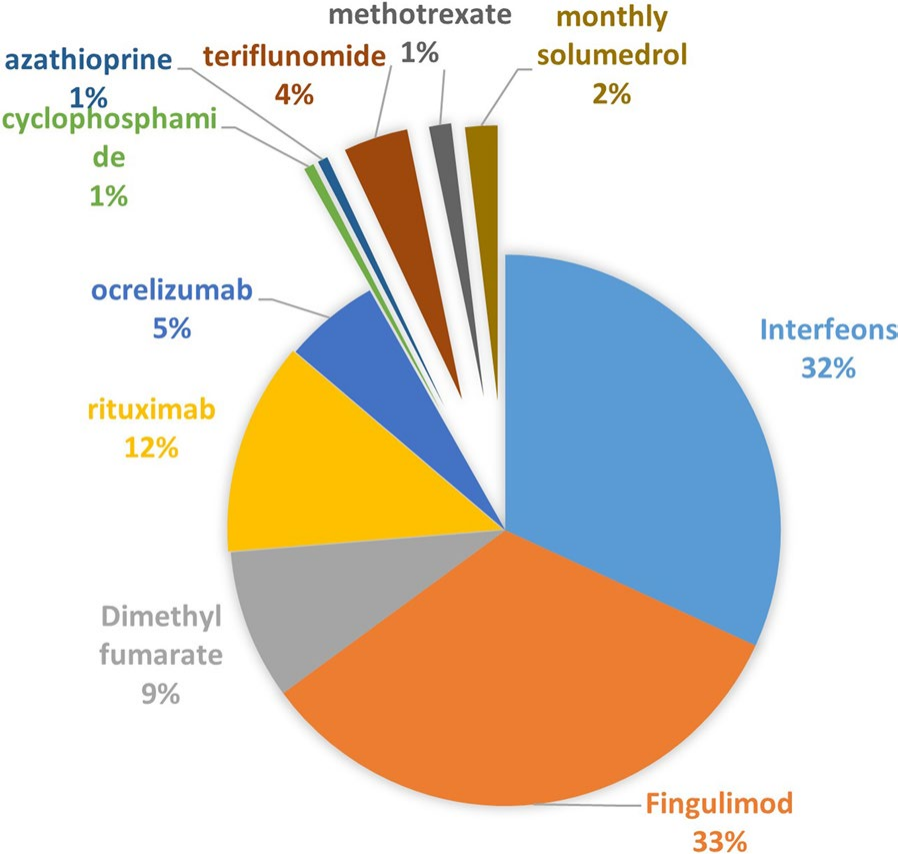
Patients’ current DMDs

This study included 160 patients whose age ranged from 17 to 49 years with a mean of (32 ± 8) years. There were 102 female patients (63.8%) and 58 male patients (36.2%). Out of 160 patients, 131 patients (81.9%) were diagnosed as RRMS, 19 patients (11.9%) were diagnosed as SPMS, 10 patients (6.2%) were diagnosed as PPMS. These patients were on various DMDs as indicated in Fig. 1

Out of 160 patients, 150 patients (93.8%) still ongoing on treatment, while 10 (6.3%) of them stopped treatment. Among patients who stopped their treatment, 8 patients stopped treatment due to lymphopenia, 1 patient stopped treatment due to shortage of drugs, and 1 patient stopped treatment due to other adverse effects from drug. No one stopped the treatment due to COVID-19 infection.

Out of 160 participants, only 39 patients (24.4%) have been infected with COVID-19 with confirmed diagnosis either by CT chest or PCR swab for COVID-19. Most of infected patients (84.6%) were treated at home with no need for hospital admission, 5 patients (12.8%) necessitated hospital admission, only 1 patient (2.6%) admitted to intensive care unit (ICU) and then discharged after recovery. The most common symptoms [5] reported by infected patients are shown in Table 1.

**Table 1:** Most common COVID-19 symptoms in MS patients

Symptom	*n*	%
General symptoms: fever, headache, malaise and anorexia, …	35	89.7
Anosmia	23	59.0
Chest manifestations: dyspnea, cough, …	20	51.3
Gastrointestinal manifestations: diarrhea, …	5	12.8
Psychiatric manifestations: depression, anxiety, …	4	10.25

Long-term manifestations [6] reported by the patients are shown in Table 2.

**Table 2:** Long-term manifestations of COVID-19 reported by MS patients

Symptom	*n*	%
No long-term manifestations	24	74.4
Sleep abnormalities	5	12.8
Headache	5	12.8
Chronic fatigue	3	7.7
Depression, anxiety	1	2.6
Vertigo	1	2.6
Anosmia	0	0.0
Peripheral neuropathy or myopathy	0	0.0

Out of 39 infected patients, 5 patients (12.8%) reported symptom suggestive of relapses after COVID infection. Out of those 5 patients, 4 patients have new lesions in brain MRI following infection.

Of note, patients were asked if there were common cold symptoms during the pandemic era and did not seek medical advice, 42 patients answered with yes, 79 patients answered with no. So if these symptoms were COVID manifestation, total infected patients may reach 50% percent of total MS patients.

### COVID-19 vaccination

**Fig. 2:**
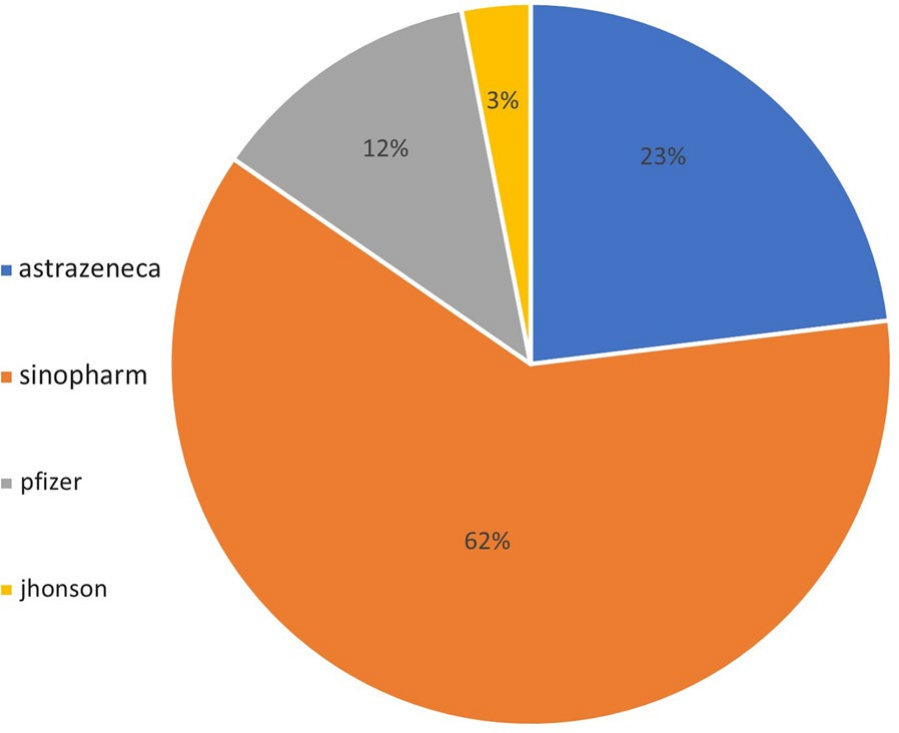
Percentage of different types of vaccines received by MS patients

Out of 160 patients, 65 patients (40.6%) were vaccinated against COVID-19, 95 patients (59.4%) still not vaccinated. Out of those 65 vaccinated patients, most of them (40 patients) received inactivated virus vaccine―Sinopharm, 15 patients received viral vector vaccine‖ AstraZeneca, 8 patients received RNA based vaccine―Pfizer and 2 patients received viral vector vaccine―Johnson (Fig. 2). Out of those vaccinated patients, 40 patients (61.54%) consulted their doctors about vaccine and 25 patients (38.46%) did not consult.

Out of the 95 unvaccinated patients, patients were asked about willingness to receive the vaccine. Thirty-three patients (34.74%) already applied for vaccination and waiting to get the vaccine, 24 patients (25.26%) were refusing to take it, 38 patients (40%) are still hesitating whether to take it or not.

Seventy-two patients (45%) got their information regarding vaccination from TV and social media, 48 patients (30%) got their information from asking their doctors, 28 patients (17.5%) got their information from their friends and 12 patients (7.5%) searched internet to know about vaccines.

Out of 65 vaccinated patients, 22 patients (33.3%) developed adverse events after vaccination, 43 patients (66.7%) did not report any adverse events. These adverse events included local manifestations at the site of injection (redness, hotness and tenderness) in 20 patients and general symptoms (fever, headache, malaise, anorexia) in 12 patients. No one reported any serious adverse events. These manifestations lasted for only days and resolved spontaneously.

Five patients (7.69%) reported exacerbations (symptoms suggestive of a relapse within 2 weeks after vaccination and not attributed to fever or infection). Three of these patients received AstraZeneca vaccine, one patient received Pfizer and one patient received Sinopharm.

## Discussion

In March 2020, WHO had declared COVID-19 as a global pandemic [7]. About 80% of people infected with SARS-CoV-2 develop a self-limiting illness, 20% require hospitalization, largely due to cardiovascular issues and about 5% require critical care and potential ventilatory support [8]. The mortality in those requiring ventilatory support is about 40–50% [9]. Many vulnerable groups are more subjected to COVID-19 infection and its severe forms, including elderly, pregnant females, people living with HIV, pediatric patients and patients receiving immunosuppressive treatments [10]. A considerable number of MS patients are treated with DMDs of which many have immunosuppressive properties. The estimated number of people with MS is 2.8 million worldwide [11]. Willis and Robertson have reported higher infection rates among MS patients with potent immune-modulator therapies [12]. It is understandable that a conservative (do no harm) approach was adopted when considering treatments, given the paucity of knowledge surrounding SARS-CoV2 biology when COVID-19 first emerged. However, it is important to recognize that the risks of poorly controlled MS may outweigh the perceived risks from COVID-19 [13].

In this study, we aimed to assess the susceptibility of MS patients for COVID-19 infection, and to assess the prevalence of COVID vaccination among MS patients. In our study, 24.3% of the patients have been infected with COVID-19 with confirmed diagnosis by CT chest or PCR swab for COVID-19. Most of infected patients (84.6%) were treated at home with no need for hospital admission, 5 patients (12.8%) necessitated hospital admission, only 1 patient (2.56%) admitted to ICU and then discharged after recovery, there was no information about mortality as the survey was retrospective. This goes with another study which observed 86 MS patients, 43 patients were COVID-19 positive by PCR. 50% had mild symptoms not requiring hospitalization, while 50% had to be hospitalized, only 3 patients were admitted to ICU among the included cohort [14].

In this study, the most common symptoms experienced by infected patients were, fever, myalgia and anorexia in 89.7% of the patients, followed by anosmia in 59% of patients, then chest symptoms as cough in 51.3% of patients. GIT symptoms as diarrhea occurred in 12.8% of patients and psychiatric manifestations as depression and anxiety in 10.25% of patients. These findings were consistent with the systematic review done by Ghayda and colleagues which concluded that fever, cough and fatigue/myalgia were the most common symptoms of COVID-19, followed by some gastrointestinal symptoms and psychiatric manifestations [15]. Regarding post-COVID prolonged symptoms, 5 (12.8%) patients reported sleep abnormalities, 5 (12.8%) patients complained of headache, 3 (7.7%) patients complained of chronic fatigue, only 1 patient complained of depression, and only 1 patient complained of vertigo. The percentage of patients in our study who developed long-term symptoms is less than reported in other studies. Lopez-Leon and colleagues concluded that 80% of the patients who were infected with SARS-CoV-2 developed one or more long-term symptoms [6]. The five most common symptoms were fatigue (58%), headache (44%), attention disorder (27%), hair loss (25%), and dyspnea (24%). But they supported our findings regarding most common symptoms. This may be due to the fact that majority of our patients had mild COVID-19 infection, so prolonged manifestations could be uncommon. Out of 39 infected patients, 5 (12.8%) patients reported symptom suggestive of relapses after COVID-19 infection (within 2 weeks of onset of symptoms). Four patients of those had new active MRI lesions. These findings were less than those reported by Parrotta and colleagues, whose study included 76 MS patients, and showed that 21.1% had neurological symptoms suggestive of a relapse. However, the author stated that neurological symptoms preceded viral symptoms by several days [16]. A case series of 7 patients with MS, reported no relapse among the including patients. Also, all patients had a self-limiting mild symptom. The authors stated that one case showed a pseudo-relapse presented by left-hand paresthesia during the respiratory symptoms, but it was interpreted by the neurologist [17]. Yet these are small samples for a conclusion.

None of the patients in this study stopped treatment due to COVID-19 infection or related cause. Growing evidence is accumulating and supporting the continuation of disease-modulating agents in MS patients. Loonstra and colleagues concluded that among the included small cohort of MS patients, there was no correlation between COVID-19 disease severity and drug discontinuation. Another study supported this evidence and concluded that there is no indication for discontinuation of DMDs among MS patients with mild symptoms during COVID-19 infection [14]. However, patients with severe respiratory symptoms and who are admitted to ICU should be advised to discontinue DMD till the resolution of respiratory symptoms [18]. There is a case series of five teriflunomide-treated MS patients who developed COVID-19 infection and continued their therapy with a self-limiting infection and without any relapse. The authors hypothesized that the immune biologic mechanisms pertaining to teriflunomide have a potential role in favoring a COVID-19-positive outcome [19].

By the end time of our study, 65 (40.6%) patients were vaccinated against COVID-19, 95 (59.4%) patients still not vaccinated. Of the vaccinated patients, 61.54% consulted their treating physician before vaccination, and 33.3% developed post-vaccination adverse events which all were self-limiting and lasted only for few days. Of the non-vaccinated patients, 34.74% were willing to be vaccinated and applied for the national registry for vaccination, while 25.26% refused to take vaccines totally, and 40.0% were hesitating to proceed for vaccination against COVID-19. A cross-sectional online survey was sent to 486 MS patients in United States and showed that 66% of the included patients were willing to receive COVID-19 vaccine, Greater willingness to receive the vaccine was associated with having a higher level of education and holding a higher perception of one's risk of catching COVID-19 [20]. A population-based study in UK showed that 94.4% were willing to receive COVID-19 vaccination, while 5.6% were refusing vaccination totally [21]. In the present study, the largest proportion (45%) of the included patients had their information about COVID-19 vaccine from TV and social media, followed by asking doctors (30%), then asking friends (17.5%), and searching internet for the least proportion (7.5%).These findings were consistent with a cross section study that reported 31.6% of the included patients accessing COVID-19 vaccine information from health care provider, 40% from social media, and 41% from family and friends [20]. While studies conducted in UK showed that source of information about COVID-19 vaccines was mainly from family in 18.5%, social media in 17.5%, and health care provider in 32.3% of the included MS patients [21]. Our data showed that MS relapse after vaccination was reported in 7.7% of the vaccinated group, 3 (60%) were after being vaccinated with viral vector "AstraZeneca", 1 (20%) after inactivated virus "Sinopharm” and last one (20%) after being vaccinated with RNA "Pfizer". In a study by Pignolo and colleauges, they concluded that available data for Pfizer vaccine in the MS population and results did not support evidence for an association between vaccination and risk for MS onset or relapses. Conversely, the risk for demyelinating events including MS onset and relapse after SARS-Cov-2 infection is higher, so immunization against SARS-COV-2 should be recommended for individuals with MS [22]. In another study on MS patients who received the 3^rd^ dose of Pfizer vaccine concluded that the most common reported adverse events were fatigue, local pain at the injection site, fever and muscle or joint pain. Transient increase in MS symptoms was reported in 3.8% of patients, none of them requiring treatment. The rate of acute relapses treated with IV steroids was 3.3% [23]. On the other hand. A case series was reported by Khayat-Khoei and colleagues, who evaluated 7 cases of MS after COVID-19 vaccination (Moderna and Pfizer), all patients developed neurological symptoms post vaccination. MRI imaging done for patients showed demyelinating lesions affecting the brain, spinal cord, and optic nerve. All patients were treated with steroids and only one required plasma exchange. All cases restored their baseline neurological status, and two patients were approaching baseline [24].

## Conclusion

Most of MS patients who had COVID-19 did not require hospitalization despite being on DMDs. The factors associated with critical illness were similar to the general at-risk patient population. MS patients were subjected to relapse after COVID-19 infection more than risk of relapse after vaccination. However, both risks were relatively low (less than 12%). There was a high willingness to receive COVID-19 vaccination among MS patients in Egypt.

## Data Availability

Available upon request from the corresponding author.

## Abbreviations

CBC: Complete blood count
CNS: Central nervous system
CRP: C-reactive protein
DMD: Disease modifying drugs
ICU: Intensive care unit
MRI: Magnetic resonance imaging
MS: Multiple sclerosis
PCR: Polymerase chain reaction
PN: Peripheral neuropathy
PPMS: Primary progressive multiple sclerosis
PwMS: Patients with multiple sclerosis
RRMS: Relapsing remitting multiple sclerosis
SPMS: Secondary progressive multiple sclerosis
UK: United Kingdom
WHO: World Health Organization
